# Is Roux-Y Binding Pancreaticojejunal Anastomosis Feasible for Patients Undergoing Left Pancreatectomy? Results from a Prospective Randomized Trial

**DOI:** 10.1155/2014/508714

**Published:** 2014-06-11

**Authors:** Anne Antila, Juhani Sand, Isto Nordback, Sari Räty, Johanna Laukkarinen

**Affiliations:** Department of Gastroenterology and Alimentary Tract Surgery, Tampere University Hospital, Teiskontie 35, P.O. Box 2000, 33521 Tampere, Finland

## Abstract

*Background*. After pancreaticoduodenectomy, the Finnish binding pancreaticojejunal anastomosis (FBPJ) seems to reduce the risk for pancreatic fistula (POPF). Our aim was to investigate whether FBPJ is feasible and prevents the risk for POPF even after left pancreatectomy (LP). *Patients and Methods*. 47 consecutive patients underwent LP. 27 patients were recruited on the basis of CT and, of these, 16 patients were randomized on the basis of findings during surgery (transection line must be left of portal vein, as 2-3 cm pancreatic mobilization is required for FBPJ) to receive either Roux-Y FBPJ or hand-sewn closure of the pancreatic remnant. *Results*. Only 34% (16/47) of the patients met the randomization criteria. Clinically significant POPF rate was higher in FBPJ group (60%) compared to thand-sewn closure group (13%; *P* < 0.05). POPF rate in FBPJ group was higher even when compared to all patients with hand-sewn closure (60% versus 37%; *P* < 0.05). Overall, FBPJ was technically feasible for only 28% of patients. *Conclusion*. FBPJ cannot be recommended for the routine closure of the pancreatic remnant after LP, as it was not technically achievable in 72% of the cases. Moreover, the technique does not seem to reduce the risk for POPF compared to the hand-sewn closure.

## 1. Introduction

Left pancreatectomy (LP) is used to treat benign and malignant lesions in the body and tail of the pancreas or after abdominal trauma. The postoperative morbidity rate remains high, 30–50% [1, 2], and this is mainly due to pancreatic fistula (POPF) resulting from leakage of pancreatic enzymes from the transsection line of the pancreas. In addition to being the most common and clinically relevant complication, POPF is often associated with other complications such as intra-abdominal abscess, delayed gastric emptying (DGE), postpancreatectomy haemorrhage (PPH), wound infection, respiratory complications, and sepsis [1]. The risk for POPF after distal pancreatectomy remains an unsolved problem despite efforts to improve the surgical resection and closure techniques of the pancreatic remnant. These include hand-sewn suture techniques, stapled closure techniques, pancreatic transsection using various energy devices, pancreaticoenteric anastomosis techniques, application of meshes, sealing with fibrin sealants, pancreatic stent placement, and administration on octreotide [3–9]. A recent retrospective cost analysis showed that patients with pancreatic fistula double the cost and dramatically increase health care resource utilization [2, 10].

Previously we have shown that after pancreaticoduodenectomy the novel Finnish binding (purse-string) pancreaticojejunal anastomosis (FBPJ) technique reduces the risk for POPF [11]. The aim of this study was to investigate whether FBPJ is a feasible technique after distal pancreatectomy and whether it prevents the risk for POPF after distal pancreatectomy.

## 2. Patients and Methods

A prospective, randomized trial was designed to include patients with the type of distal pancreatic resection that is technically possible with FBPJ (RPT arm). In addition, all pancreatic distal resections were included in the prospective follow-up (PFU arm).

### 2.1. Surgical Technique

**Figure 1:**
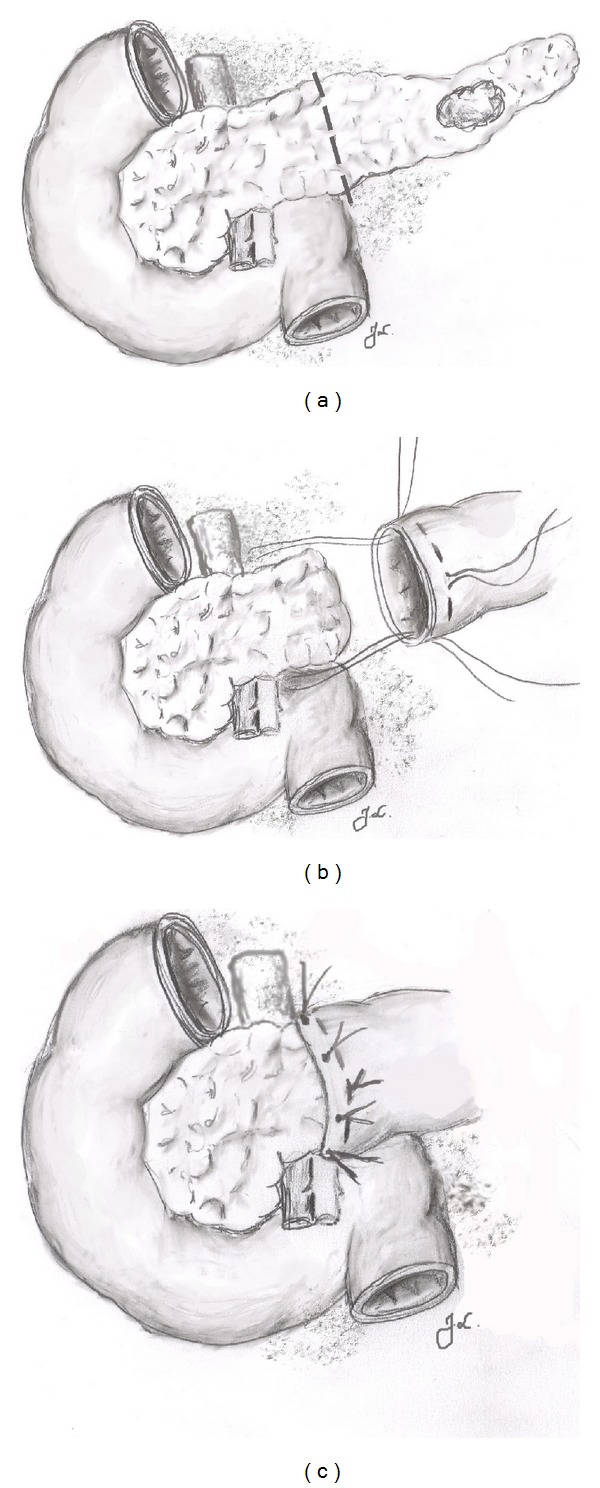
Schematic drawing of the binding (purse-string) pancreaticojejunal anastomosis (FBPJ) after left pancreatectomy. The transsection line needs to be clearly to the left of the portal vein (a). The pancreatic remnant is mobilized 2-3 cm and it is inserted inside the jejunal loop with the aid of anchoring sutures (b). The purse string applied in the jejunum is tightened to secure the anastomosis (c).

In FBPJ, the pancreatic remnant was inserted 2-3 cm inside the jejunal limb with the aid of seven peripancreatic sutures (4-0 Maxon, Covidien, USA) after which the purse-string suture (4-0 PDS, Ethicon, USA) was tightened and a roux-Y entero-enteroanastomosis was performed (Figure 1). In the hand-sewn closure group, the main pancreatic duct was closed by suturing, followed by oversewing the pancreatic stump with 4-0 Maxon. A Penrose drain was placed near the anastomosis in all patients. A schematic drawing of the FBPJ is shown in Figure 1.

### 2.2. Recruitment Criteria for the RPT Arm

FBPJ is technically achievable only when the transection line of pancreas is clearly to the left of the portal vein because the pancreatic remnant needs to be mobilized 2-3 cm to be able to insert it into the jejunal limb. All patients were studied preoperatively by contrast-enhanced computer tomography scan (CT). Patients eligible for randomization according to the location of tumour in the CT analysis were recruited for the study. The rest of the patients were included in the prospective follow-up.

### 2.3. Randomization Criteria for the RPT Arm

After removing the distal pancreas, the patients still considered eligible for the FBPJ (i.e., transection line to the left of the portal vein) were randomized to receive either FBPJ or traditional hand-sewn closure of the pancreatic stump.

### 2.4. Patient Care and Follow-Up

Perioperatively all patients received a single-dose antibiotic prophylaxis IV (ceftriaxone 2 g, Rocephalin, Roche, Finland, and metronidazole 500 mg, metronidazole, Brown, Germany) and routine antithrombotic (enoxaparin 40 mg, Klexane, Sanofi-Aventis, France, or tinzaparin 4500 IU, Innohep, LEO Pharma, France) prophylaxis s.c. Postoperatively the patients were monitored by the standard pancreatic resection protocol of Tampere University Hospital. Abdominal drain output was recorded daily and the amylase concentration was measured from it on the third postoperative day, and thereafter if the drain still remained in place. The drain was removed when the drain amylase output was less than three times the serum upper limit. The urine trypsinogen strip test was used to detect postoperative pancreatitis and was measured daily during the first postoperative week [12]. Patient demographics (age, sex, BMI, and comorbidities) were compared and postoperative complications (fistulas, bleeding, abscesses, and wound infections) and mortality were defined and compared between the groups. POPF was classified into three grades (A, B, and C) depending on the clinical impact according to the ISGPF classification [13].

### 2.5. Power Analysis

For the RPT arm, population size was estimated on the basis of the results from our earlier study of FBPJ after pancreaticoduodenectomy [11], where the rate of clinically relevant (grades B-C) POPF was reduced by 50% compared to our historical controls. If the patients with hand-sewn closure had twice as much clinically relevant POPF compared to FBPJ (30% versus 15%), we would need 26 patients in each group to be able to show a statistically significant difference with power *π* = 0.80 (*α* 0.05). We estimated that about one-third of the patients would not meet the recruitment criteria based on CT and that about 10% of the recruited patients would not meet the randomization criteria according to findings during surgery. Thus for 52 randomized patients we would need 58 recruited patients, and for those we would need a population of 78 distal pancreatectomies. We planned to run the interim analysis when 29 patients had been recruited and estimated that about 40 distal pancreatectomies would be needed to achieve this recruited population.

The interim analysis was run in August, 2013. A total of 47 consecutive patients (16 M/31 F) had undergone distal pancreatectomy with the remaining pancreatic head in Tampere University Hospital between October 2009 and July 2013. We were prepared to increase our series but this proved unnecessary after analysing the results of these 47 patients.

The study protocol was approved by the Ethics Committee of Tampere University Hospital. The study was registered with clinical.trials.com NCT02113046.

Statistical analysis was performed using Fisher's exact test, Mann-Whitney *U*-test, and logistic regression test. *P* < 0.05 was considered statistically significant.

## 3. Results

**Figure 2:**
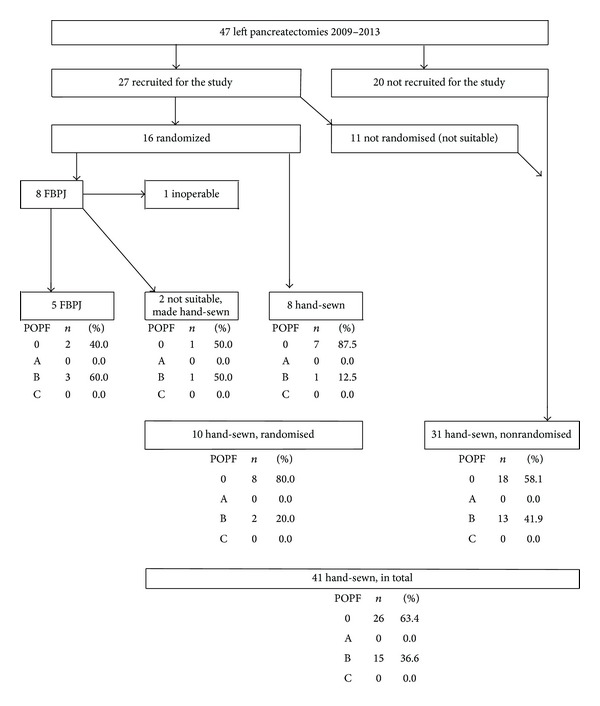
Flow chart of the study patients and POPF rate in each group. Out of 47 consecutive patients, 27 were recruited and only 16 of these met the randomization criteria. Finally, only 5 patients received a FBPJ (POPF 60%) and 8 patients a hand-sewn closure (POPF 12.5%) from the randomized patients. The POPF rate was 36.6% in all hand-sewn closure patients.

Out of the 47 caudal resections, 27 met the recruitment criteria, but only 16 of these met the randomization criteria in the operation (as described in Section 2, the transsection line or the pancreas needed to be clearly to the left of the portal vein for the patient to be randomized). Patients were randomized into FBPJ or hand-sewn group. Out of the 8/16 patients randomised for FBPJ, in two patients, FBPJ was still technically impossible to accomplish and they received a hand-sewn closure. In addition, one had after all an advanced disease, and distal pancreatectomy was not performed. 8/16 were randomized for hand-sewn closure. Thus, of the recruited patients, five received a FBPJ and ten a hand-sewn closure in the RPT arm and 11 in the non-RPT arm. More 20 patients received a hand-sewn closure in the prospective follow-up arm. Thus a total of 41 patients had a hand-sewn closure. The flow chart is shown in Figure 2.

Patients were well comparable for age, sex, and comorbidities. Patient demographics are shown in Table 1. Indications for surgery were malignant tumours in 28 patients, benign tumors in 14 patients, chronic pancreatitis in 1 patient, and pancreatic pseudocyst in 3 patients. The final histopathological diagnoses are shown in Table 2.

The main endpoints of the study were the feasibility of FBPJ in LP patients and the POPF rate. POPF was significantly higher in the FBPJ group, in which 3/5 patients (60%) developed a grade B POPF compared to the hand-sewn group, where 1/8 patients (13%) developed a grade B fistula (*P* < 0.05). In the FBPJ group two patients had an operatively placed drain removed and needed an interventional radiology placed drain due to subsequent abscess. The third patient had a high amylase output from the operatively placed drain, which was kept in place and removed five weeks postoperatively. In the hand-sewn group the patient who developed a grade B fistula was discharged with the drain but was readmitted and the CT showed pancreatitis and collection of fluid. The operatively placed drain was removed after six weeks, after which no additional drainage was needed. Fistula rates are shown in Figure 2.

The fistula rate in the FBPJ group was significantly higher, not only compared to the RPT hand-sewn group (POPF gr B 60% versus 13%; *P* < 0.05) but also compared to all hand-sewn closures (POPF gr B 60% versus 37%; *P* < 0.05).

In addition to the high fistula rate, only 13/47 (27%) of patients were eligible for FBPJ according to our interim analysis, so we decided to discontinue the study at this point.

30-day mortality was zero. There was no postoperative haemorrhage. No reoperation was needed in either group. Among the prospective follow-up hand-sewn patients, four patients had a wound infection, one patient had a lymphatic leak, and two patients had pancreatitis. Urine trypsinogen strip test was positive on two or more days in one patient in FBPJ (20%) and in ten patients in all hand-sewn groups (24%; NS) suggesting postoperative pancreatitis. Blood loss during surgery, length of hospital stay, and readmission rate to hospital were comparable between the groups. All these characteristics are shown in Table 1.

## 4. Discussion

POPF remains the most common complication after distal pancreatectomy with an incidence between 20 and 40% [3, 14, 15] and many surgical techniques for resection and closure of the pancreatic remnant have been studied without major success [3–5, 7, 9, 14, 16–19]. We have previously shown that the novel FBPJ technique reduces the risk for pancreatic fistula after pancreaticoduodenectomy [11], and within this study we investigated whether the FBPJ technique was feasible even for LP. We concluded that FBPJ cannot be recommended for a routine for pancreatic remnant closure after LP, as it is not technically achievable in most of the cases and does not seem to reduce the risk for POPF compared to the hand-sewn closure.

Stapler and suture closure are the two most common strategies for managing the pancreatic remnant. In the DISPACT trial [3], which included 450 patients, two groups of patients were randomized to either stapler or hand-sewn closure of the pancreatic remnant with no difference found in POPF incidence. The meta-analysis likewise revealed no significant differences between suture and stapler closure [4]. Several other methods have also been tried [16]. Recently the use of saline-coupled radiofrequency dissector in stump closure reduced the POPF rate, but further prospective studies are needed [5]. Pancreaticojejunostomies (PJ) have also been performed to reduce the fistula rate and the findings have been encouraging [6, 8]. In 2007 Wagner et al. [6] found a zero POPF rate Roux-en-Y end-to-side PJ after suture closure versus 20% in suture closure only. In their study, POPF was not classified into three grades according to the ISGPF definition and the number of patients was only 23 versus 20 in either group. In 2013 Meniconi et al. [8] reported a retrospective analysis where the fistula rate was also zero in PJ and 29% in the hand-sewn group. In the PJ group the main pancreatic duct was closed, after which the pancreatic remnant was invaginated into a jejunal loop. This was a nonrandomized retrospective study on a small group of patients (24 versus 12). We have shown previously that after pancreaticoduodenectomy the novel FBPJ technique reduces the risk for pancreatic fistula [11].

In this study we wanted to investigate whether FBPJ can also be used in distal pancreatectomy and whether it reduces the risk of pancreatic fistulae. FBPJ is technically achievable only when the transsection line of the pancreas is clearly to the left of the portal vein because the pancreatic remnant needs to be mobilized 2-3 cm before it can be inserted inside the jejunal loop. This is the reason why only 27 out of 47 patients who received an LP resection were recruited. We estimated the suitable patients based on the location of the tumour preoperatively with the help of contrast-enhanced CT scan. Randomization was done intraoperatively and only 16 patients out of the total 46 met the randomization criteria, and of these one had an inoperable tumour and in two the FBPJ was impossible to perform. In most of the distal pancreatectomies it is not technically possible to mobilize the pancreatic remnant 2-3 cm in order to insert it inside the jejunal loop. The FBPJ would therefore have been technically feasible for only 28% (13/47) of patients. In the other studies where PJ was performed with good results [6, 8] the pancreatic remnant was invaginated instead of being inserted inside the jejunal loop. The anastomosis was made by capsule-to-seromuscular single layer sutures when the pancreatic remnant did not need to be mobilized as in our FBPJ technique. This may explain why it was possible to perform PJ on all patients in those studies.

FBPJ did not decrease the number of pancreatic fistulae in this small study. On the contrary, it seemed to increase the cases of POPF. In addition, FBPJ anastomosis is feasible in only a minority of patients, which is why we discontinued the study after performing the interim analysis. The number of patients who received FBPJ was small, but, as most patients did not seem to be eligible for this kind of anastomosis, it was challenging to achieve a large enough patient population in the FBPJ group to show the differences in the fistula forming.

In conclusion, the FBPJ technique, which reduces the POPF rate after pancreaticoduodenectomy, is suitable only for selected patients with LP and thus it cannot be recommended for routine use in the closure of the pancreatic remnant. In addition, according to this study it does not seem to reduce the risk of POPF.

## Figures and Tables

**Table 1:** Patient demographics and postoperative complications in the groups (FBPJ: randomized binding pancreaticojejunal group, hand-sewn rand.: randomized hand-sewn group, and hand-sewn all: all patients with hand-sewn anastomosis).

	FBPJ		Hand-sewn rand.		Hand-sewn all	
*n*	5		8		41	
Age (median and range)	67	(55–74)	60	(26–80)	66	(26–85)
Gender M/F	1/4		2/6		15/26	
BMI (mean)	28.2		27.2		26	
Smoking	1	(20%)	0		7	(17%)
Alcohol abuse (audit > 6)	0		1	(12.5%)	5	(12.1%)
Diabetes	0		2	(25%)	5	(12.1%)
Cardiac disease	0		1	(12.5%)	3	(7.3%)
Hypertension	2	(40%)	2	(25%)	20	(48.7)
Wound infection	0		0		4	(9.7%)
PPH	0		0		0	
Abscess	3	(60%)	0		9	(21.9)
Pancreatitis (CT verified)	0		1	(12.5%)	2	(4.9%)
Trypsinogen strip test positive	1	(20%)	1	(12.5%)	10	(24.3%)
Length of stay (days)	10	(7–15)	7	(6–9)	7	(6–32)
Readmission	1	(20%)	1	(12.5%)	4	10%
Operative time (mins, median, and range)	170	(136–300)	162	(115–200)	170	(90–305)
Blood loss (mL, median, and range)	750	(300–2350)	750	(300–1300)	750	(100–3600)
Mortality	0		0		0	

**Table 2:** Final histopathologic diagnoses (FBPJ: randomised binding pancreaticojejunal group, hand-sewn rand.: randomised hand-sewn group, and hand-sewn all: all patients with hand-sewn anastomosis).

	FBPJ		Hand-sewn rand.		Hand-sewn all	
*n*	5		8		41	
Adenocarcinoma	2	(40%)	4	(50%)	13	(32%)
Neuroendocrine tumour	3	(60%)	2	(25%)	9	(22%)
Intraductal papillary mucinous neoplasm					4	(10%)
Pseudocyst			1	(12.5%)	3	(7%)
Mucinous cystic neoplasm					2	(5%)
Chr. pancreatitis					1	(2%)
Haemangioma			1	(12.5%)		
Nesidioblastoma					1	(2%)
Kidney ca metastases					1	(2%)
Serous cystadenoma					5	(12%)
None					1	(2%)
